# The efficacy of immunosuppressive drugs induction therapy for lupus nephritis: a systematic review and network meta-analysis

**DOI:** 10.1080/0886022X.2023.2290365

**Published:** 2023-12-12

**Authors:** Yongqiang Dong, Jinmin Shi, Shanshan Wang, Yanhong Liu, Shirong Yu, Lijun Zhao

**Affiliations:** a College of Pharmacy; Department of Pharmacy, Renmin Hospital, Hubei University of Medicine, Shiyan, China; b Laboratory of Chinese Herbal Pharmacology, Department of Pharmacy, Renmin Hospital; Biomedical Research Institute; Hubei Key Laboratory of Wudang Local Chinese Medicine Research, Hubei University of Medicine, Shiyan, China; c College of Plant Science and Technology, Innovation Academy of International Traditional Chinese Medicinal Materials, National-Regional Joint Engineering Research Center in Hubei for Medici Hubei University of Medicinal Plant Breeding and Cultivation, Huazhong Agricultural University, Wuhan, China; d Medicinal Plant Engineering Research Center of Hubei Province, Institute for Medicinal Plants, Huazhong Agricultural University, Wuhan, China

**Keywords:** Lupus nephritis, voclosporin, leflunomide, azathioprine, cyclosporine A, tacrolimus, mycophenolate mofetil, cyclophosphamide, belimumab

## Abstract

**Objective:**

This study was to assess the safety and effectiveness of immunosuppressive agents, specifically Voclosporin, when used in conjunction with mycophenolate mofetil (MMF) induction therapy for the management of lupus nephritis (LN).

**Methods:**

A systematic review and network meta-analysis (NMA) was conducted on randomized controlled trials investigating the efficacy of immunosuppressant-induced therapy for LN. The random effects model was used in the analysis. I^2^ was used to evaluate the heterogeneity of the model. Odds ratios (OR) and 95% credible intervals (CrI) were computed to assess and compare the relative effectiveness and safety of various treatment protocols.

**Results:**

The study included a total of 16 randomized controlled trials (RCTs) involving 2444 patients with LN. The analysis results indicated that there was no significant difference in terms of partial remission (PR) between the drugs. However, when considering complete remission (CR), the combination of Voclosporin with MMF showed the highest remission rate, followed by Tacrolimus (TAC). Unfortunately, Voclosporin in combination with MMF had the highest risk of infection and serious infection, indicating a lower safety profile.

**Conclusions:**

Voclosporin in combination with MMF demonstrated the highest efficacy as an induction therapy for LN. However, it should be noted that the risk of infection and serious infection was found to be high with this regimen. On the other hand, TAC not only showed efficacy but also had a lower risk of infection and serious infection, making it a favorable option in terms of safety. This study did’ not include results on other adverse events.

## 1. Introduction

Systemic lupus erythematosus (SLE) is a significant autoimmune disease that affects the kidneys in around 60% patients. Lupus nephritis (LN) is the primary cause of morbidity and mortality related to renal involvement in SLE patients, making it the most severe complication of organ involvement in SLE [1–3]. Approximately 40% of SLE patients develop LN. If not promptly and appropriately treated, renal function in SLE patients will progressively worsen, leading to increased morbidity and mortality [4]. Therefore, a key objective in SLE treatment is to control the impairment of renal function caused by LN. The treatment of LN typically involves two phases: an induction phase and a maintenance phase [5].

Immunosuppressive drugs and glucocorticoids have been found to be effective in treating LN, leading to improved renal function and reduced mortality rates [2,6]. However, it is important to note that these treatment options can also suppress the immune system, making infections a significant concern for LN patients [7,8]. For instance, Cyclophosphamide (CYC) is commonly used as the standard treatment for Grade III/IV LN, but its long-term use can increase the risk of serious infections and ovarian toxicity [9]. Despite the availability of therapeutic drugs like CYC and MMF, the incidence of renal remission in LN patients remains low [10]. The safety and efficacy of three drugs, MMF, IVCY, and TAC, in treating patients with LN were compared in a network meta-analysis. This study found that TAC was the most effective drug with the lowest risk of serious infection [11]. Voclosporin, when combined with MMF, showed good efficacy in clinical trials for treating LN, but it still had a relatively high risk of infection [12,13]. To compare the safety and efficacy of different immunosuppressive agents, this study used network meta-analysis to compare the relative efficacy and safety of Voclosporin combined with MMF and other immunosuppressive agents for treating LN. Due to the strong emphasis of the 2023 EULAR recommendation on the use of Belimumab or voclosporin as an additional therapy in addition to standard immunosuppressants for the treatment of active lupus nephritis (LN), Belimumab has been added to our study.

## 2. Materials and methods

### 2.1. Search strategy

Our systematic evaluation and network meta-analysis followed the recommendations of AHRQ and the Cochrane Handbook. The reporting of results adhered to the PRISMA guidelines **[**14,15]. Since our study only involved the analysis of published results, review board approval was not required. We registered our study protocol with PROSPERO under registration number CRD42023437668. We conducted a search in the Web of Science, PubMed, and Embase databases, retrieving documents up to 30 October 2023. The search used keywords such as Lupus nephritis, Voclosporin, Leflunomide, Azathioprine (AZA), Cyclosporine A (CSA), Tacrolimus (TAC), Mycophenolate Mofetil (MMF), Cyclophosphamide (CYC) and Belimumab. The results were limited to Randomized Controlled Trials (RCTs). Duplicate literature was removed using Endnote.

### 2.2. Eligibility criteria

#### 2.2.1. Population

This study focused on patients with lupus nephritis grades III, IV, or V, either alone or in combination with grades III or IV. The study included patients of all ages and genders, the language used in the study was restricted to English.

#### 2.2.2. Interventions and comparator

This study included randomized controlled trials (RCTs) of Voclosporin, Leflunomide, AZA, CSA, TAC, MMF, CYC and Belimumab for LN. Subgroup analysis of RCTs included in this study was excluded.

#### 2.2.3. Outcomes

The primary outcome indicators of effectiveness included the number of patients in complete renal remission (CR) and the number of patients in partial renal remission (PR). Primary indicators of safety included the number of patients who experienced an infection and the number of patients who experienced a serious infection.

### 2.3. Screening and abstraction process

The title and abstract of the articles were reviewed independently by two trained abstractors (AO, AB), who then selected the PDFs for download. Two independent excerpts (AO, AB) extracted information from the literature and entered it into a Microsoft Excel spreadsheet. Articles deemed potentially relevant to this study will undergo full-text review. And discrepancies will be resolved through group discussion. A table was created to extract the last name of the first author, publication time, lupus nephritis classification, sample size, and treatment drug. The relative effectiveness of drug therapy was assessed based on the number of patients who achieved CR or PR, while the relative safety was measured by the number of patients who experienced an infection or serious infection. Odds ratios (OR) and 95% confidence intervals (CrI) were used to compare the relative effectiveness and safety.

### 2.4. Risk of bias

We utilized the Corcoran risk assessment tool to perform a risk assessment of the articles included in our study. We evaluated various indicators, such as the generation of random sequences, concealment of allocation, blinding of participants and personnel, blinding of evaluators for outcomes, incomplete data for outcomes, selective reporting, and other sources of potential bias. Each criterion was categorized as presenting a low risk, high risk, or ambiguous bias risk. Ambiguous bias risk indicates a lack of information or uncertainty regarding potential bias. A study is considered to have a low risk of bias when all criteria are rated as low. If at least one criterion is rated as having an ambiguous risk but there is no high risk identified, the study is classified as having a risk of ambiguous bias. Conversely, a study is deemed to have a high risk of bias when at least one criterion is classified as high-risk [16]. The assessment of quality was conducted independently by two authors (AO, LZ), and disagreements were resolved through intergroup discussion.

### 2.5. Calculations and statistical analysis

A random effects model was constructed using the gemtc and rjags packages in R (4.3.1) for each outcome in a Bayesian framework with a network meta-analysis [17], following the guidance of Harrer et al. [18]. The convergence of the algorithm to prior distributions was evaluated by assessing the convergence of four chains using Brooks Gelman Rubin statistics. The trajectory density map was also used to evaluate the convergence degree of the model. Convergence was considered achieved if the potential scale reduction factor (PSRF) was less than 1.05. The consistency of the model was evaluated using the node segmentation method. When *p*-value > 0.05, it indicates that the consistency of the model results is relatively good. The degree of freedom of the model in this study was the number of experiments minus one. Node splitting was only implemented in certain treatment schemes due to the lack of direct comparison in the network. The relative efficacy and safety of each therapeutic agent were compared using odds ratio (OR) and 95% credible interval (CrI). The overall ranking of safety and efficacy was expressed using the surface under the cumulative ranking (SUCRA), which ranged from 0.00 to 1.00. No assessment of publication bias was performed due to the limited number of studies (less than 10) per treatment pair. The GRADE method was utilized to assess the confidence level of relevant outcomes in comparison to specific drugs in network meta-analysis. This helps determine the level of evidence for CR, PR, infection, and serious infection. We considered the risk of bias, inconsistency, indirectness, and publication bias.

## 3. Results

### 3.1. Study cohort characteristics

**Figure 1.**
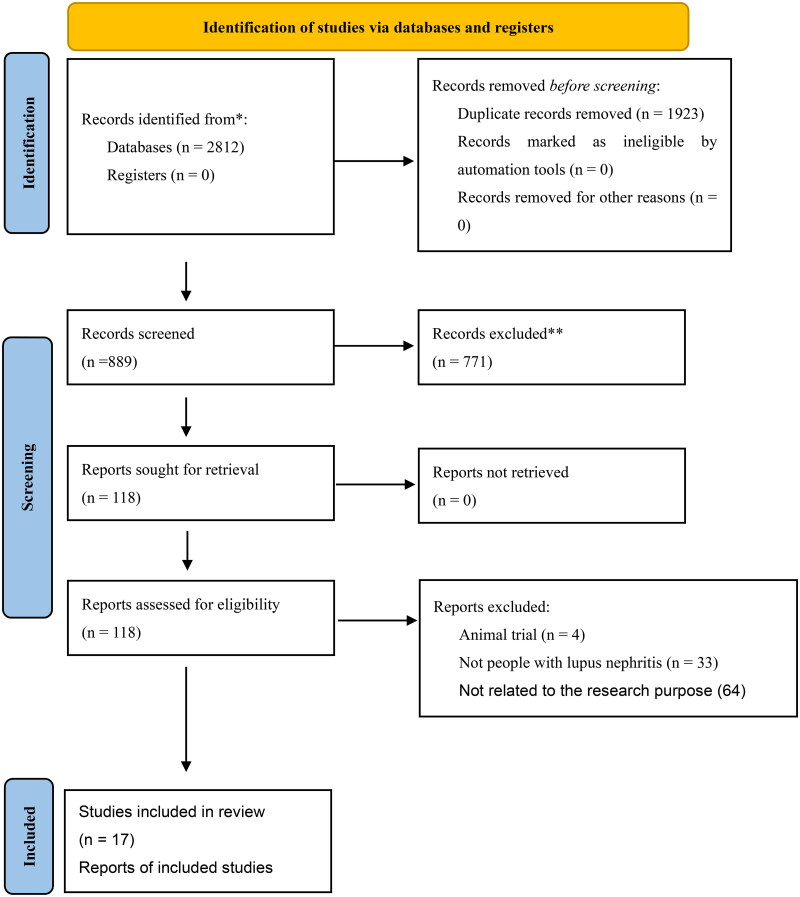
Flow diagram of study identification and selection.

**Figure 2.**
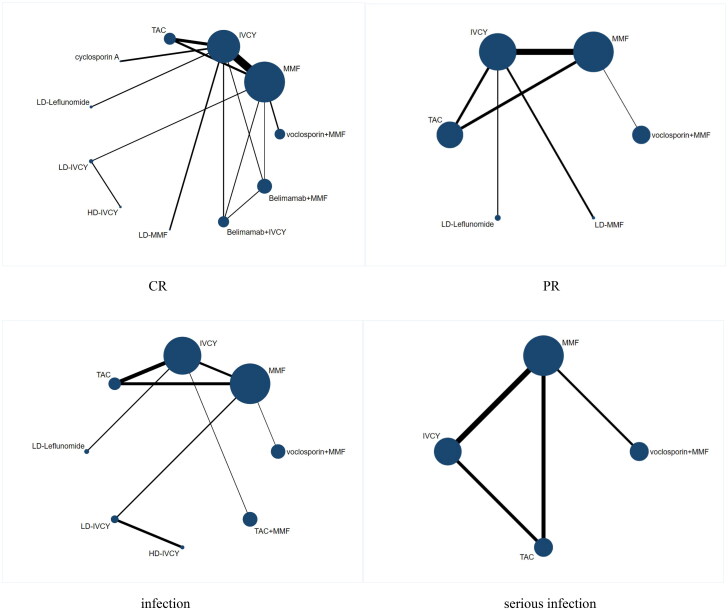
Network diagram for complete remission (CR), partial remission (PR), infection events and serious infection events. The size of each node is proportional to the sample size of the individual treatment regimen; the widths of the connecting lines are proportional to the number of studies compared between the two regimens.

**Figure 3.**
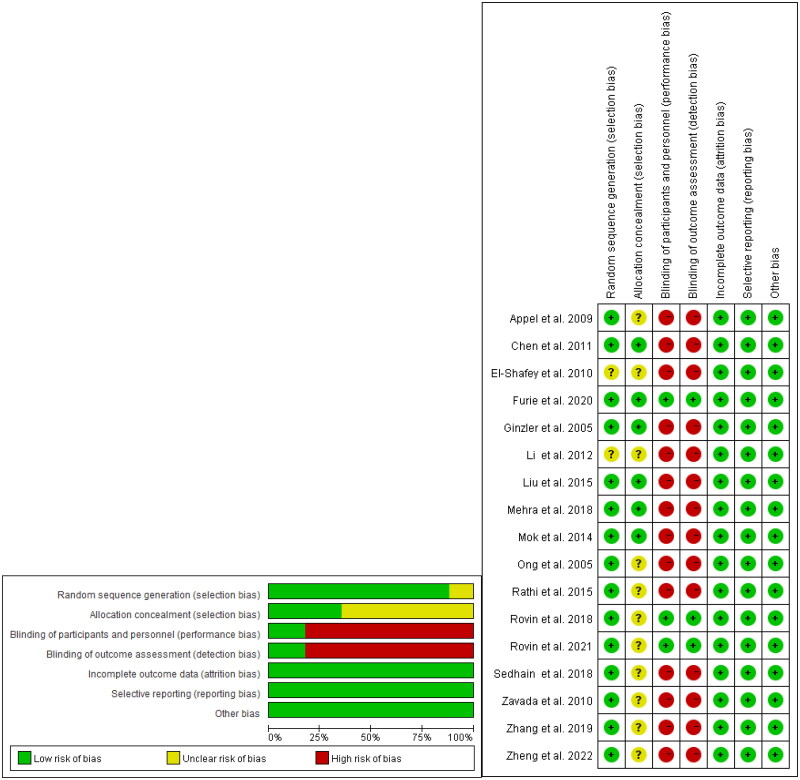
Bias risk assessment table for articles included in the study.

A total of 17 randomized controlled trials (RCTs) were included in this study, involving 2890 patients with LN [10,12,13,19–32]. However, data on infection and serious infection were only partially available due to differences in the statistical approach used in each study. Only the literature on induction therapy was analyzed in this study. The screening and inclusion process of the studies is shown in Figure 1. The basic characteristics of the included studies are presented in Table 1. Figure 2 illustrates a network diagram depicting the relationships between Complete renal response (CR), Partial renal response (PR), infection, and serious infection. A total of 15 (88%) studies demonstrated a low risk of bias during the generation of random sequences. Additionally, 6(35%) studies showed a low risk of bias in their allocation concealment. 3 (18%) studies provided reports on the allocation treatment that indicated blindness to both participants and investigators. Similarly, 3 (18%) studies reported blind outcome evaluation. Furthermore, all studies exhibited a low risk of bias when reporting incomplete outcomes, selective outcomes, and other potential sources of bias (Figure 3). Table 2 presented the GRADE evaluation of the outcome indicators for the treatment plan. It is worth noting that most of the studies compared the effectiveness of MMF and intravenous cyclophosphamide (IVCY), as well as IVCY and TAC.

**Table 1.** Characteristics of included studies in the network meta-analysis.

study	Sample size	Biopsy class	Treatments drugs
Zheng et al. 2022 (19)	299	III, IV, V (single or combined III or IV)	TAC vs. IVCY
Rovin et al. 2021 (12)	357	III, IV, V (single or combined III or IV)	voclosporin + MMF vs. MMF
Furie et al. 2020 (20)	446	III, IV, V (single or combined III or IV)	IVCY vs. Belimamab + IVCY MMF vs. Belimamab + MMF
Zhang et al. 2019 (21)	100	III, IV, V (single or combined III or IV)	LD-Leflunomide vs. IVCY
Sedhain et al. 2018 (22)	42	III, IV, V (single or combined III or IV)	LD-MMF vs. IVCY
Mehra et al. 2018 (23)	75	III, IV	LD-IVCY vs. HD-IVCY
Rovin et al. 2018 (13)	177	III, IV, V (single or combined III or IV)	voclosporin + MMF vs. MMF
Liu et al. 2015 (24)	362	III, IV, V (single or combined III or IV)	TAC + MMF vs. IVCY
Rathi et al. 2015 (25)	100	III, IV, V (single or combined III or IV)	LD-IVCY vs. MMF
Mok et al. 2014 (26)	150	III, IV, V (single or combined III or IV)	MMF vs. TAC
Li et al. 2012 (27)	60	III, IV, V (single or combined III or IV)	MMF vs. TAC vs. IVCY
Chen et al. 2011 (28)	81	III, IV, V (single or combined III or IV)	TAC vs. IVCY
Zavada et al. 2010 (29)	40	III, IV	IVCY vs. cyclosporin A
El-Shafey et al. 2010 (30)	47	III, IV	MMF vs. IVCY
Appel et al. 2009 (31)	370	III, IV, V (single or combined III or IV)	MMF vs. IVCY
Ong et al. 2005 (32)	44	III, IV	IVCY vs. MMF
Ginzler et al. 2005 (33)	140	III, IV, V	MMF vs. IVCY

*LD* low dose, *MMF* Mycophenolate mofetil, *IVCY* Intravenous Cyclophosphamide, *TAC* Tacrolimus.

**Table 2.** Summary of confidence in network therapy evaluation of immunosuppressive therapy inducing remission of lupus nephritis.

Outcome & Treatment Strategy	Confidence in Evidence	Reasons for Downgrading Confidence in Evidence
Complete renal response		
voclosporin + MMF	High ●●●●	No downgrades in confidence
Belimamab + MMF	High ●●●●	No downgrades in confidence
Belimamab + IVCY	High ●●●●	No downgrades in confidence
MMF	Low ●●○○	Downgrade 2 levels in confidence based on Risk of bias (-1] and Publication bias (-1]
IVCY	Low ●●○○	Downgrade 2 levels in confidence based on Risk of bias (-1] and Publication bias (-1]
TAC	Low ●●○○	Downgrade 2 levels in confidence based on Risk of bias (-1] and Publication bias (-1]
LD-IVCY	Low ●●○○	Downgrade 2 levels in confidence based on Risk of bias (-1) and Publication bias (-1)
LD-Leflunomide	Low ●●○○	Downgrade 2 levels in confidence based on Risk of bias (-1)and Publication bias (-1)
HD-IVCY	Low ●●○○	Downgrade 2 levels in confidence based on Risk of bias (-1) and Publication bias (-1)
LD-MMF	Low ●●○○	Downgrade 2 levels in confidence based on Risk of bias (-1) and Publication bias (-1)
cyclosporin A	Low ●●○○	Downgrade 2 levels in confidence based on Risk of bias (-1) and Publication bias (-1)
Partial renal response		
voclosporin + MMF	High ●●●●	No downgrades in confidence
MMF	Low ●●○○	Downgrade 2 levels in confidence based on Risk of bias (-1) and Publication bias (-1)
IVCY	Low ●●○○	Downgrade 2 levels in confidence based on Risk of bias (-1) and Publication bias (-1)
TAC	Low ●●○○	Downgrade 2 levels in confidence based on Risk of bias (-1) and Publication bias (-1)
LD-Leflunomide	Low ●●○○	Downgrade 2 levels in confidence based on Risk of bias (-1) and Publication bias (-1)
HD-IVCY	Low ●●○○	Downgrade 2 levels in confidence based on Risk of bias (-1) and Publication bias (-1)
LD-MMF	Low ●●○○	Downgrade 2 levels in confidence based on Risk of bias (-1) and Publication bias (-1)
LD-IVCY	Low ●●○○	Downgrade 2 levels in confidence based on Risk of bias (-1) and Publication bias (-1)
Infection		
voclosporin + MMF	High ●●●●	No downgrades in confidence
MMF	Low ●●○○	Downgrade 2 levels in confidence based on Risk of bias (-1) and Publication bias (-1)
TAC	Low ●●○○	Downgrade 2 levels in confidence based on Risk of bias (-1) and Publication bias (-1)
IVCY	Low ●●○○	Downgrade 2 levels in confidence based on Risk of bias (-1) and Publication bias (-1)
LD-Leflunomide	Low ●●○○	Downgrade 2 levels in confidence based on Risk of bias (-1) and Publication bias (-1)
HD-IVCY	Low ●●○○	Downgrade 2 levels in confidence based on Risk of bias (-1) and Publication bias (-1)
TAC + MMF	Low ●●○○	Downgrade 2 levels in confidence based on Risk of bias (-1)and Publication bias (-1)
Serious infection		
voclosporin + MMF	High ●●●●	No downgrades in confidence
MMF	Low ●●○○	Downgrade 2 levels in confidence based on Risk of bias (-1) and Publication bias (-1)
TAC	Low ●●○○	Downgrade 2 levels in confidence based on Risk of bias (-1) and Publication bias (-1)
IVCY	Low ●●○○	Downgrade 2 levels in confidence based on Risk of bias (-1) and Publication bias (-1)
HD-IVCY	Low ●●○○	Downgrade 2 levels in confidence based on Risk of bias (-1) and Publication bias (-1)

1. The credibility of evidence was evaluated based on the GRADE (Recommended Evaluation, Development, and Evaluation Scale) standard, which was based on risk of bias, Inconsistency, Indirection and Publication bias.

### 3.2. Network meta-analysis of the efficacy of Voclosporin, Leflunomide, AZA, CSA, TAC, MMF and CYC in RCTs

We evaluated the efficacy of different treatment drugs by analyzing the CR and PR rates.

In terms of CR, the CR rate of IVCY was found to be lower than that of TAC (OR = 0.2453, 95% CrI = 0.07631 − 0.7751), and significantly lower than that of Belimamab + MMF (OR = 0.08164, 95% CrI = 0.01459 − 0.4453). The CR rate was higher in MMF than in IVCY (OR = 3.761, 95% CrI = 1.265 − 12.61). The CR rate of voclosporin in combination with MMF was much higher than that of MMF(OR = 6.516, 95%CrI = 1.608 − 25.49), Belimamab + MMF (OR = 11.53, 95%CrI =1.067 − 151.8), IVCY (OR = 24.84, 95% CrI = 4.289 − 153.1) and low-dose Leflunomide (OR = 41.01, 95% CrI = 1.764 − 917.5) (Table 3 A). In terms of PR, there was no significant difference in the comparison between different treatment drugs.

**Table 3.** Significant differences in treatment of lupus nephritis in CCR, PCR, infection and severe infection. **A** CR. OR > 1 indicates better treatment effect

Treatment	Reference	OR (95 % CrI)
voclosporin + MMF	MMF	6.515 (1.815, 23.92)
voclosporin + MMF	IVCY	19.11 (3.88, 104)
voclosporin + MMF	LD-Leflunomide	31.31 (1.712, 718.4)
voclosporin + MMF	Belimamab + IVCY	11.53 (1.067, 151.8)
MMF	IVCY	2.951 (1.13, 8.08)
IVCY	TAC	0.27 (0.08686, 0.8005)
IVCY	Belimamab + MMF	0.08164 (0.01459, 0.4453)

**Table:** **B** infection. OR > 1 indicates a higher risk of infection

Treatment	Reference	OR (95% CrI)
MMF	TAC	9.941 (1.116, 89.24)

**Table:** **C** serious infection. OR > 1 indicates a higher risk of infection

Treatment	Reference	OR (95% CrI)
IVCY	TAC	6.435 (1.016, 55.02)

### 3.3. Network meta-analysis of the safety of Voclosporin, Leflunomide, AZA, CSA, TAC, MMF and CYC in RCTs

The risk of infection in MMF was significantly higher compared to TAC (OR = 9.941, 95% CrI = 1.16-89.24) (Table 3B). Similarly, the risk of serious infection in IVCY was significantly higher than in TAC (OR = 6.435, 95% CrI = 1.016, 55.02) (Table 3C).

### 3.4. Result sorting

Table 4 presents the SUCRA values (%) for the effectiveness and safety of each treatment option. Voclosporin in combination with MMF ranked the highest in terms of CR, closely followed by Belimamab + MMF and high-dose IVCY. For PR, once again, voclosporin in combination with MMF ranked the highest, with low-dose Leflunomide and low-dose MMF following closely behind. When considering the risk of infection, voclosporin in combination with MMF had the highest ranking, indicating the highest risk for patients. Low-dose IVCY had the second highest risk, while TAC had the lowest risk of infection. Similarly, when analyzing serious infections, voclosporin combined with MMF showed the highest risk, while TAC exhibited the lowest risk.

**Table 4.** Rank probability of efficacy and safety for each treatment drug.

Treatments	Complete remission	Partial remission	Infection	severe infection
voclosporin + MMF	91.97%	79.58%	75.98%	72.94%
MMF	58.51%	25.08%	60.07%	64.94%
IVCY	23.93%	30.60%	39.55%	58.73%
TAC	60.52%	29.55%	11.48%	3.39%
cyclosporin A	35.88%	/	/	/
LD-Leflunomide	19.33%	70.12%	39.75%	/
LD-IVCY	46.15%	/	73.69%	/
HD-IVCY	82.27%	/	50.10%	/
LD-MMF	31.43%	65.07%	/	/
TAC + MMF	/	/	49.37%	/
Belimamab + IVCY	37.54%	/	/	/
Belimamab + MMF	82.98%	/	/	/

‘/’ means that this treatment plan was not included in this analysis.

### 3.5. Subgroup analysis

Our analysis revealed heterogeneity in the results of complete renal response when comparing MMF and IVCY. Four studies, namely Appel et al. [10], Ong et al. [31], El-Shafey et al. [30], and Li et al. [27], were found to have unknown risks. Consequently, we grouped these four studies with unknown risks and performed a subgroup analysis. The results of our analysis indicated an I2 value of 0, suggesting no heterogeneity among these four studies. Among them, the first two studies had unknown risks due to allocation concealment, while the last two had unknown risks related to the generation of random sequences and allocation concealment. Therefore, we concluded that the heterogeneity might be attributed to improper random allocation of patients.

## 4. Discussion

In this network meta-analysis, we systematically collected and evaluated existing evidence on the relative efficacy and safety of immunotherapy-induced therapy in patients with LN. Our research findings indicated that the combination of voclosporin and MMF achieved the highest complete remission (CR) rate, followed by Belimamab + MMF, HD-IVCY and TAC, both of which were superior to LD-Leflunomide. When Belimamab was combined with MMF for the treatment of lupus nephritis, its treatment ranking was very high, second only to voclosporin + MMF. However, when Belimamab was combined with CYC, the treatment ranking was very low. It is important to note that the combination of voclosporin and MMF carried a higher risk of infection and serious infection, making it less safe. On the other hand, TAC emerged as the safest drug option due to its lower risk of infection and serious infection.

Our findings align with previous meta-analyses. In a network meta-analysis comparing the safety and efficacy of MMF, IVCY, and TAC for treating patients with LN, Lee et al. [11] discovered that TAC not only demonstrated efficacy but also had the lowest risk of serious infection. Similarly, Li et al. [33] conducted a network meta-analysis comparing the efficacy of rituximab and common induction therapy for LN and found that TAC was equally effective with a low risk of infection. Our research results also support these findings. We found that the combination of voclosporin and MMF provided the most effective treatment option, but it carried the highest risk of infection and serious infection, and had low safety. TAC, on the other hand, ranked second in terms of efficacy, demonstrating not only good effectiveness but also the lowest risk of infection and serious infection, along with good safety. It is important to note that infection is the primary cause of death in SLE patients [34]. Our findings indicated that the combination of voclosporin and MMF posed the highest risk of infection among the various immunosuppressants studied. However, it is important to note that this increase in risk did not reach statistical significance. On the other hand, the risk of infection associated with TAC was lower than that of MMF, and the risk of serious infection was lower than that of IVCY. Both these differences were statistically significant. In conclusion, patients with LN require long-term maintenance therapy even after achieving renal remission. Therefore, TAC and voclosporin in combination with MMF are recommended as the preferred treatment options for patients with LN. In terms of safety, we believe that TAC is the safest immunosuppressant when considering the risks of infection and serious infection.

Caution should be exercised when interpreting the results of our study due to several limitations. Firstly, the number of Randomized Controlled Trials (RCTs) included in the studies was small, and some studies had relatively small sample sizes. Secondly, our study did not have RCTs for direct comparisons, as network meta-analysis is not a substitute for such comparisons. Thirdly, the heterogeneity of the experimental design and patient characteristics in the included RCTs may have influenced the results of our network meta-analysis. Factors such as dosing regimen, administered dose, and timing of experimental endpoints can all impact the outcomes. Lastly, our evaluation of the drug’s safety and efficacy focused solely on the number of patients achieving complete response (CR), partial response (PR), and those experiencing infection and serious infection. Therefore, our results do not provide a comprehensive assessment of the drug’s efficacy and safety.

Nevertheless, this network meta-analysis had multiple advantages. The studies included in our analysis were randomized controlled trials and were evaluated using the Jadad scale. Only a few studies were of low quality, while the majority of the RCTs were of high quality. Despite some studies having small sample sizes, our pooled analysis involved a total of 2444 patients with LN. By combining the results from various studies, the study provides more accurate data, enhancing the statistical validity and resolution.

## 5. Conclusions

In conclusion, our study found that TAC not only demonstrated excellent efficacy but also had a lower risk of infection and serious infection when compared to other immunosuppressants. However, we can only provide a low level of confidence in the efficacy evaluation of TAC. Therefore, our results should be interpreted with caution. On the other hand, the high quality of evidence suggested that the combination of voclosporin and MMF yielded the most favorable as therapeutic effect. However, it is important to note that this combination also carried a higher risk of infection, including serious infections, and had a relatively lower safety profile Similarly, high-quality evidence suggested that the therapeutic effect of Belimamab combined with MMF was second only to voclosporin + MMF. It is important to note that this network meta-analysis did not provide conclusive evidence regarding other adverse events.
